# Personalized Prediction of Short- and Long-Term PTH Changes in Maintenance Hemodialysis Patients

**DOI:** 10.3389/fmed.2021.704970

**Published:** 2021-09-14

**Authors:** Markus Pirklbauer, David A. Bushinsky, Peter Kotanko, Gudrun Schappacher-Tilp

**Affiliations:** ^1^Department of Internal Medicine IV – Nephrology and Hypertension, Medical University Innsbruck, Innsbruck, Austria; ^2^Department of Medicine, University of Rochester School of Medicine, Rochester, NY, United States; ^3^Renal Research Institute New York, New York, NY, United States; ^4^Icahn School of Medicine at Mount Sinai, New York, NY, United States; ^5^Institute for Mathematics and Scientific Computing, University of Graz, Graz, Austria; ^6^Institute of Electronic Engineering, FH Joanneum-University of Applied Sciences, Graz, Austria

**Keywords:** precision medicine, secondary hyperparathyroidism, parathyroid hormone, patient-level prediction model, hemodialysis

## Abstract

**Background:** Personalized management of secondary hyperparathyroidism is a critical part of hemodialysis patient care. We used a mathematical model of parathyroid gland (PTG) biology to predict (1) short-term peridialytic intact PTH (iPTH) changes in response to diffusive calcium (Ca) fluxes and (2) to predict long-term iPTH levels.

**Methods:** We dialyzed 26 maintenance hemodialysis patients on a single occasion with a dialysate Ca concentration of 1.75 mmol/l to attain a positive dialysate-to-blood ionized Ca (iCa) gradient and thus diffusive Ca loading. Intradialytic iCa kinetics, peridialytic iPTH change, and dialysate-sided iCa mass balance (iCaMB) were assessed. Patient-specific PTG model parameters were estimated using clinical, medication, and laboratory data. We then used the personalized PTG model to predict peridialytic and long-term (6-months) iPTH levels.

**Results:** At dialysis start, the median dialysate-to-blood iCa gradient was 0.3 mmol/l (IQR 0.11). The intradialytic iCa gain was 488 mg (IQR 268). Median iPTH decrease was 75% (IQR 15) from pre-dialysis 277 to post-dialysis 51 pg/ml. Neither iCa gradient nor iCaMB were significantly associated with peridialytic iPTH changes. The personalized PTG model accurately predicted both short-term, treatment-level peridialytic iPTH changes (r = 0.984, *p* < 0.001, *n* = 26) and patient-level 6-months iPTH levels (r = 0.848, *p* < 0.001, *n* = 13).

**Conclusions:** This is the first report showing that both short-term and long-term iPTH dynamics can be predicted using a personalized mathematical model of PTG biology. Prospective studies are warranted to explore further model applications, such as patient-level prediction of iPTH response to PTH-lowering treatment.

## Introduction

Secondary hyperparathyroidism (sHPT) is a common sequela of chronic kidney disease (CKD), with intact PTH (iPTH) levels typically starting to rise once the glomerular filtration rate drops below approximately 45 ml/min. Most patients with CKD stage 5 present with sHPT. sHPT is a consequence and key mediator of CKD mineral bone disorder (CKD-MBD) and linked to disturbed calcium (Ca) and phosphate homeostasis, renal osteodystrophy, vascular and valvular calcification, erythropoietin hypo-responsiveness, and cardiovascular morbidity and mortality (1–3). The pathogenesis of sHPT is multifactorial, it involves FGF23-mediated reduction of active vitamin D levels and decrease of serum ionized Ca (iCa). The iCa decline causes parathyroid gland (PTG) cells to increase PTH release and synthesis and to proliferate in response to reduced stimulation of vitamin D (VDR) and Ca-sensing (CaSR) receptors. Hyperphosphatemia and blunted FGF23-mediated inhibition of PTH synthesis further aggravate sHPT as CKD progresses (4, 5). The resulting PTG changes are classified into four stages: diffuse hyperplasia, early nodularity, nodular hyperplasia, and single nodular gland. Initial stages are characterized by polyclonal PTG cell proliferation. At later stages, the PTG cells become increasingly monoclonal and show a concomitant reduction of VDR and CaSR expression. These processes perpetuate sHPT and render the PTG more and more refractory to medical therapy (e.g., VDR agonists, calcimimetics) (6–8). On a population level, sHPT stages are associated with altered parameters of mineral metabolism (Ca, phosphate, PTH) and CKD stage (9, 10). On a patient level, the observed heterogeneity of individual disease characteristics complicates the clinical assessment of PTG status and the prediction of drug effects (11). There is an unmet clinical need for novel approaches to characterize sHPT progression more accurately. To gain insight into the complex PTG biology, we developed and recently published a physiology-based mathematical model of PTG biology (12). In this study we validate the individualization of this mathematical model by predicting the patients' short-term peridialytic iPTH changes in response to diffusive intradialytic Ca loading. We then used the model to predict the level of iPTH at 6-months using only readily available clinical data, such as iCa and phosphate measurements, iPTH history, dialysis vintage and information about vitamin D therapy.

## Materials and Methods

### Clinical Data

This research utilized biochemical measurements, intradialytic iCa kinetics, and iCa mass balance (iCaMB) data from 28 previously studied maintenance hemodialysis (HD) patients (13). In this study, patients received one dialysis treatment with a dialysate Ca concentration of 1.75 mmol/l to attain a positive dialysate-to-blood iCa gradient and diffusive Ca loading. The Fresenius FX100 dialyzer was used for HD treatment. Two patients received cinacalcet before iCaMB assessment and were excluded from this study. Dialysate-sided iCaMB assessment is described in detail elsewhere (13). Long-term clinical and laboratory data (iCa, phosphate, and iPTH), as well as medication, was obtained from patients' electronic health records over a 6-months period before and after iCaMB assessment, respectively. iPTH (1–84) was measured using an electrochemiluminescence immunoassay (Roche Diagnostics, Mannheim, Germany) in a certified lab (Central Institute for Medical and Chemical Laboratory Diagnostics; Medical University Innsbruck, Austria).

### Mathematical Model

The mathematical model of PTG biology is described in detail elsewhere (12) (see also Supplementary Material). Briefly, the model considers two cell populations, cells in the quiescent state, which can undergo apoptosis or proliferate, and cells in the secretory active state. The model captures fast PTH responses, e.g., the release of pre-formed and stored PTH within seconds to minutes as a response to a drop in iCa. Moreover, the model predicts long-term alterations of PTG biology, such as a decreased intracellular PTH degradation rate, increased PTH production rate, and increased cellular proliferation rate. In order to individualize the PTG model and allow for patient specific short and long-term iPTH predictions we estimate key model parameters (i.e., individual optimal iCa and phosphate levels, PTG gland size, intracellular degradation rate, PTH production rate, and PTH clearance rate) from individual patients' longitudinal data (i.e., dialysis vintage as well as calcitriol therapy and all available iCa, phosphate and iPTH concentrations within 6 months before calcium mass balance assessment) (see also Supplementary Material for details).

We use the PTG model with patient specific model parameters to:

1. validate individual model parameters by simulating short-term iPTH dynamics during dialysis based on intradialytic iCa changes.
2. predict long-term iPTH under different conditions, such as a rise or drop of either iCa or phosphate over the next 6 months.
3. validate long-term iPTH predictions by using available iCa and phosphate measurements over 6 months as input parameters to predict the iPTH level measured closest to 180 days post iCaMB assessment. The median of predicted iPTH values in the week of actual iPTH measurement was used for calculating the correlation between predicted and measured iPTH values since iCa and phosphate measurements were not necessarily available at the same day. Data sets for this validation include iCa and phosphate concentrations until 180 days post iCaMB assessment as well as the reference iPTH around 180 days post iCaMB assessment. Patients on calcimimetic treatment or kidney transplantation within 180 days post iCaMB assessment were excluded from the analysis. From the initial 26 patients, 13 either received kidney transplantation, calcimimetic treatment, moved to another HD facility or died during the 180 days post iCaMB assessment. Complete validation data sets, thus, were available in 13 patients.

### Statistical Analysis

Baseline characteristics and iCa kinetic data are presented as median values or frequencies. Associations among intradialytic iCa kinetic parameters and peridialytic iPTH change were assessed by simple linear correlation analysis. Kendall's Tau-b coefficients were computed for non-parametric correlation of metric variables. Pearson regression analysis was used to assess the correlation between measured and predicted iPTH concentrations. In addition, we present Bland Altman plots for the difference between measured and predicted iPTH values. Statistical analysis was performed with SPSS version 24.0 and Matlab (Mathworks). The level of significance (*p-*value) was set to 0.05.

### Statement of Ethics

The study was conducted in accordance with the World Medical Association Declaration of Helsinki. The study protocol was approved by the Innsbruck Medical University ethics committee prior to study initiation (protocol number AN2014-0313 343/4.7 391/5.3). Written informed consent was obtained prior to study inclusion from each participant using a standardized information and consensus form. Data collection was conducted using a standardized evaluation form (case report form) according to GCP recommendations. All patient associated samples and clinical data are subject to privacy protection according to the current European General Data Protection Regulation. Patient information was managed entirely coded. This study complies with the STROBE standards for reporting of observational studies.

## Results

We utilized long-term clinical and laboratory data, intradialytic iCa kinetics, and peridialytic iPTH levels of 26 maintenance HD patients (see Table 1 for patient baseline characteristics).

**Table 1:** Baseline characteristics of maintenance hemodialysis patients (*n* = 26).

**Parameter**	**Median**	**Min**	**Max**	**IQR**
Female/male (%)	35/65			
Age (years)	65	19	84	19
Dialysis vintage (days)	218	26	4479	429
Clinical dry weight (kg)	69.8	52.6	106	29.4
Dialysis treatment time (min)	240	180	240	0
Ultrafiltration volume (ml)	1,726	0	4,224	2,045
Total serum Ca, average of last 6 months (mmol/l)	2.2	1.91	2.47	0.18
Serum phosphate, average of last 6 months (mmol/l)	1.78	1.05	2.08	0.52
iPTH, average of last 6 months (pg/ml)	326	49	554	240
Alkaline phosphatase, average of last 6 months (U/l)	87	47	329	65

*Ca, calcium; iPTH, intact parathyroid hormone; IQR, interquartile range*.

### Intradialytic iCa Mass Balance and Peridialytic iPTH Change

**Figure 1:**
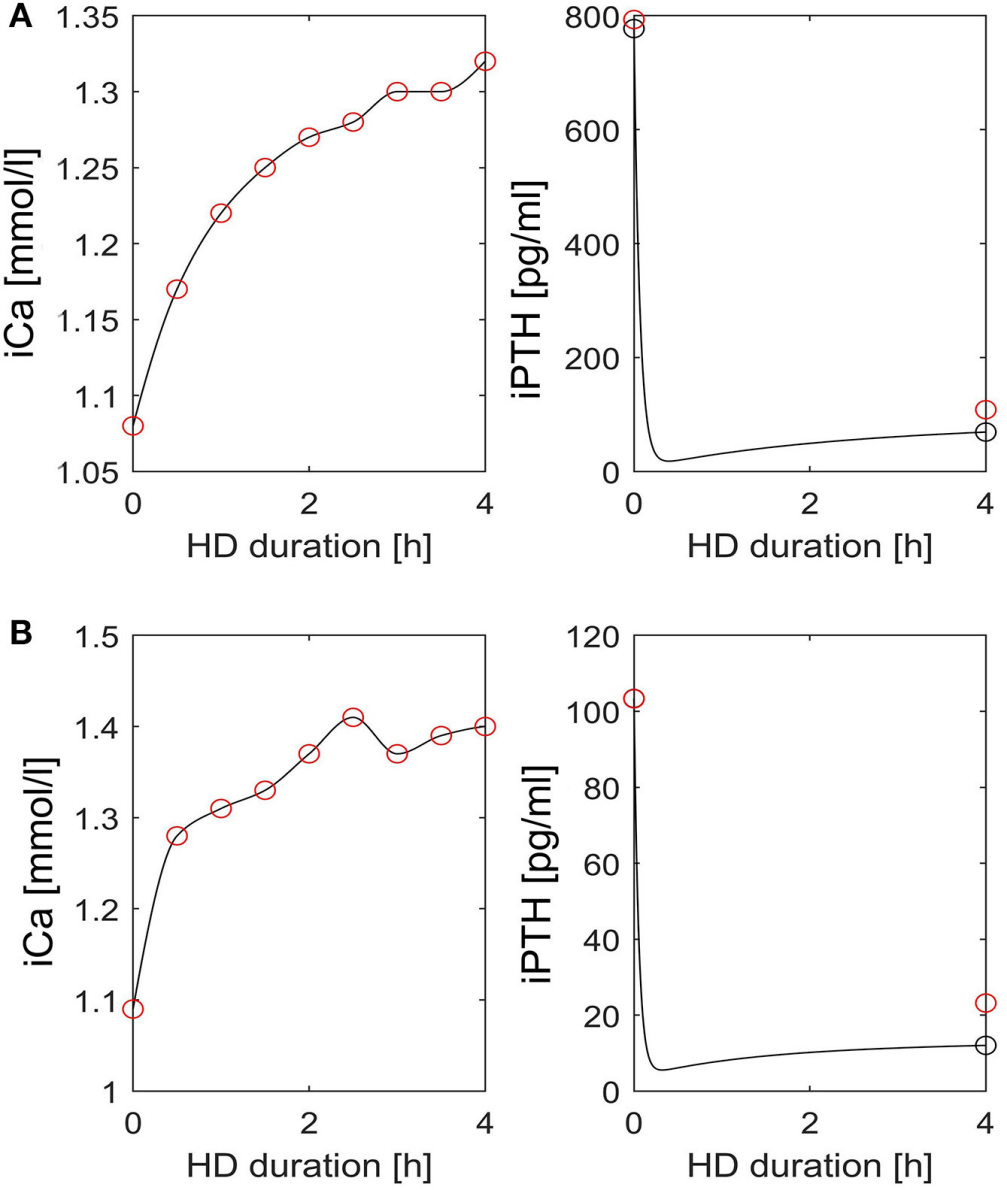
Intradialytic iCa and iPTH kinetics in two patients. **(A,B)** Left: Interpolated iCa (black line) is based on measured iCa concentrations (red circles). Right: iPTH dynamics (black line) as predicted by the patient specific PTG model. The red circles indicate measured iPTH levels pre- and post-dialysis, respectively. The black circles indicate simulated iPTH levels pre- and post-dialysis, respectively. HD, hemodialysis; iCa, ionized calcium; iPTH, intact parathyroid hormone.

**Figure 2:**
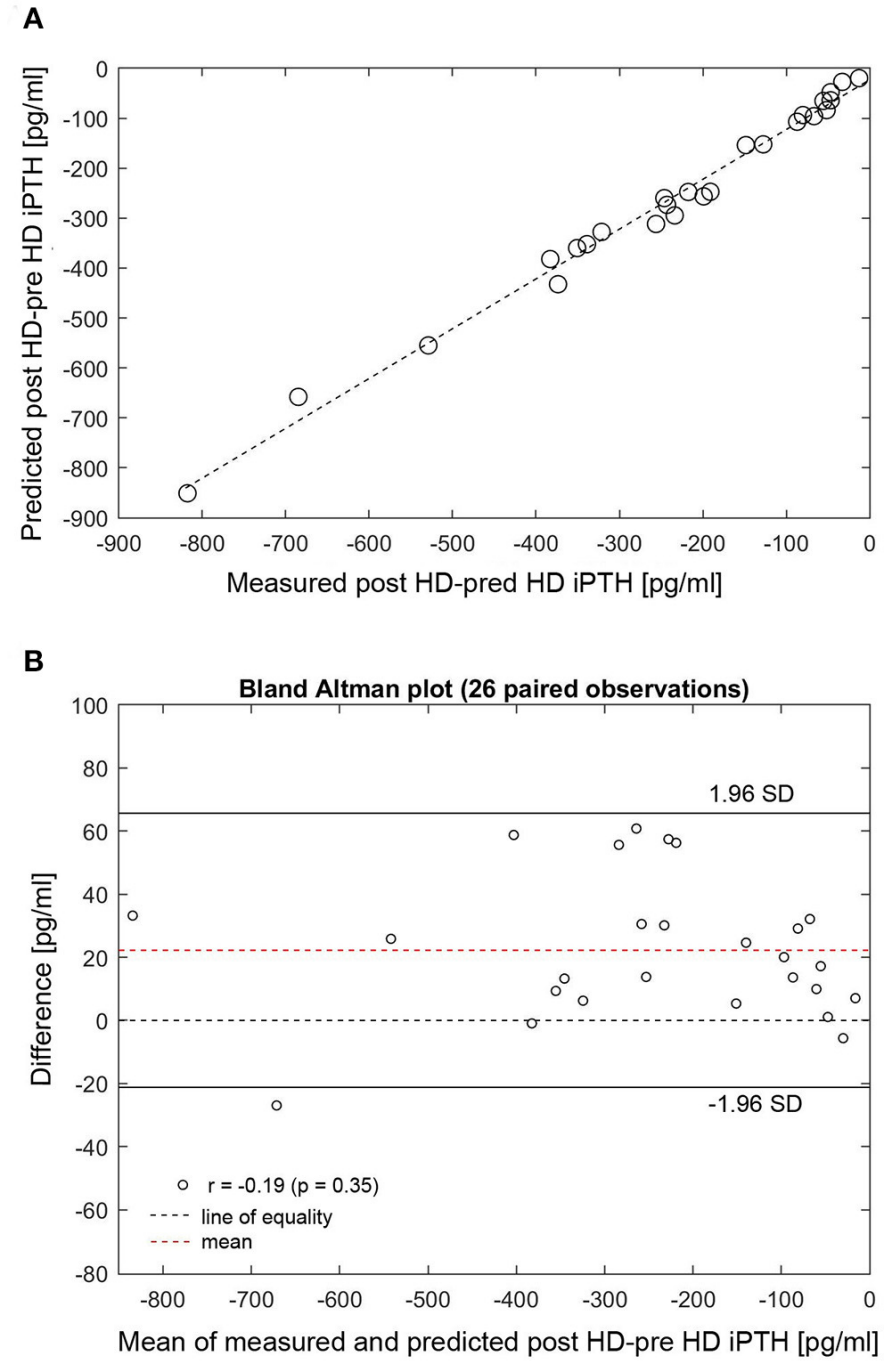
**(A)** Correlation between measured and predicted post-minus-pre HD iPTH (*n* = 26). (**B)** Bland Altman plot for differences between measured and predicted post-minus-pre HD iPTH. **(A)** Measured (x-axis) and predicted (y-axis) peridialytic iPTH change (r = 0.984, *p* < 0.001). The peridialytic change was calculated as post-HD iPTH minus pre-HD iPTH levels. HD, hemodialysis; iPTH, intact parathyroid hormone; SD, standard deviation.

At dialysis start, the median dialysate-to-blood iCa gradient was 0.3 mmol/l (IQR 0.11). The iCaMB was 488 mg/HD (IQR 268). Median iPTH decrease was 75% (IQR 15) from 277 to 51 pg/ml (Table 2). Neither the iCa gradient (b = 0.129, *p* = 0.354) nor iCaMB (b = 0.031, *p* = 0.826) correlate with peridialytic iPTH change. Two examples of individual intradialytic iCa kinetics are depicted in Figures 1A,B. The two patients had comparable iCa at HD start (1.08 and 1.09 mmol/l, respectively) and post-to-pre dialysis iCa ratios (1.22 and 1.28, respectively). However, their peridialytic iPTH changes differ significantly (−684.8 and −80.12 pg/ml). Nevertheless, the personalized PTG model accurately predicted post-dialysis iPTH levels in both patients. Across all 26 patients, measured and predicted peridialytic iPTH changes were highly correlated (r = 0.984; *p* < 0.001) (Figure 2A) thus the PTG model accurately predicts treatment-level peridialytic iPTH change. Furthermore, the Bland Altman plot for differences between measured and predicted iPTH showed no proportional bias (r = −0.19, *p* = 0.35, the lower and upper limits of agreement are −21.2 and 65.6 pg/ml, respectively) (Figure 2B).

**Table 2:** Intradialytic iCa kinetics, iCa mass balance, and peridialytic iPTH change (*n* = 26).

**Parameter**	**Median**	**Min**	**Max**	**IQR**
Serum iCa pre HD (mmol/l)	1.17	1.01	1.31	0.08
Serum iCa post HD (mmol/l)	1.35	1.26	1.49	0.06
iCa gradient (dialysate/serum) (mmol/l)	0.3	0.15	0.56	0.11
Delta iCa (mmol/l)	0.19	0.08	0.34	0.09
iCa mass balance (mg)	488	170	765	268
iPTH pre HD (pg/ml)	277	37	898	291
iPTH post HD (pg/ml)	51	12	115	54
Peridialytic iPTH change (% of pre)	−75	−35	−92	15

*HD, hemodialysis; iCa, ionized calcium; iPTH, intact parathyroid hormone; IQR, interquartile range*.

### Long-Term iPTH Dynamics

**Figure 3:**
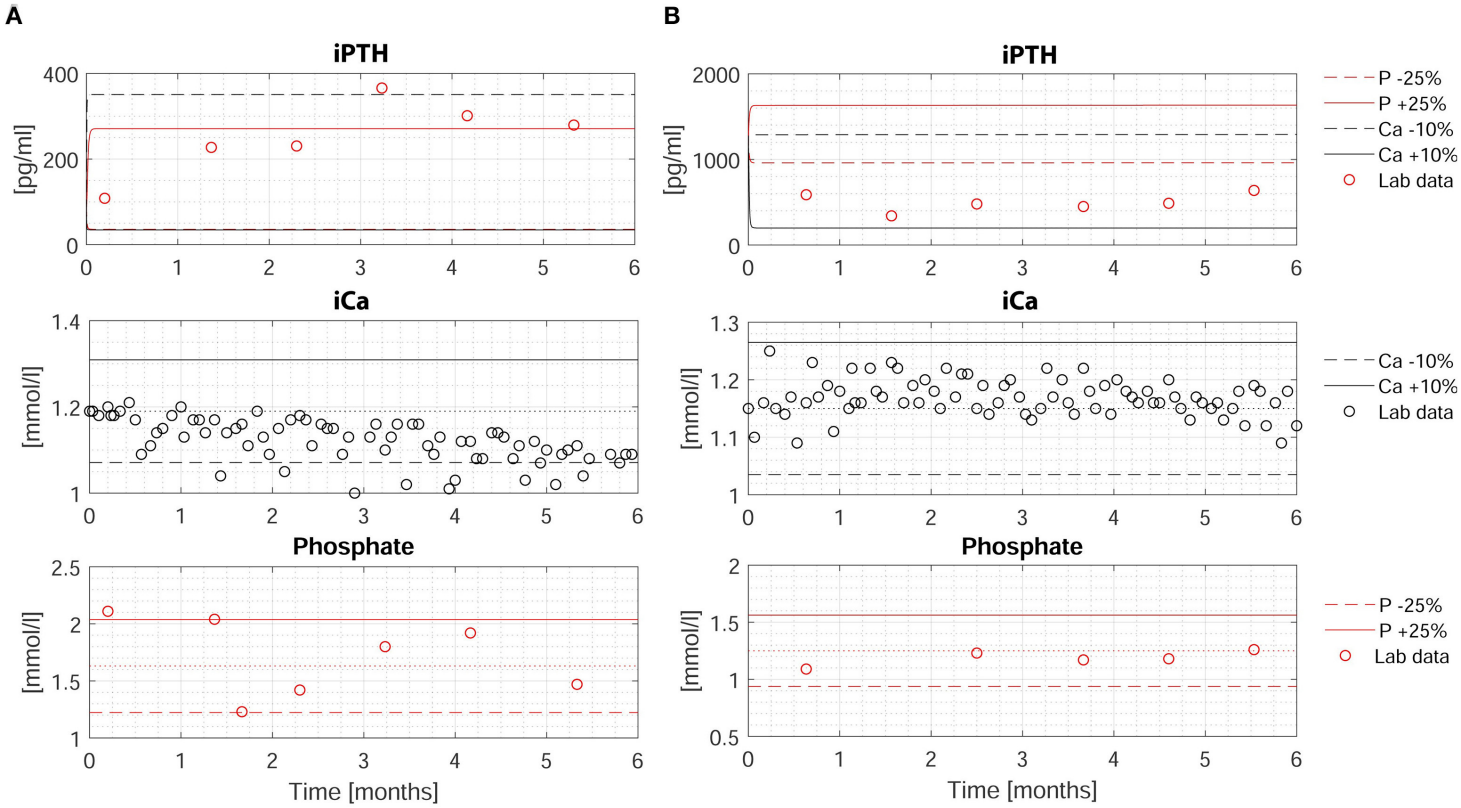
Measured and predicted iPTH levels over 6-months period in two patients. **(A,B)** Upper: Measured (red circles) and predicted (dashed and solid lines) iPTH levels based on four different scenarios: (i) a decrease of phosphate by 25% (dashed red line); (ii) an increase of phosphate by 25% (solid red line); (iii) a decrease of iCa by 10% (dashed blue line); (iv) an increase of iCa by 10% (solid blue line). Middle: Measured iCa (black circles). The dotted line represents the baseline iCa concentration at the time of iCa mass balance assessment. The solid and dashed line represent +10% and −10% of the baseline value, respectively. Lower: Measured phosphate (red circles). The dotted line represents the baseline phosphate concentration at the time of iCa mass balance assessment. The solid and dashed line represent +25% and −25% of the baseline value, respectively. HD, hemodialysis; iCa, ionized calcium; iPTH, intact parathyroid hormone; P, phosphate.

**Figure 4:**
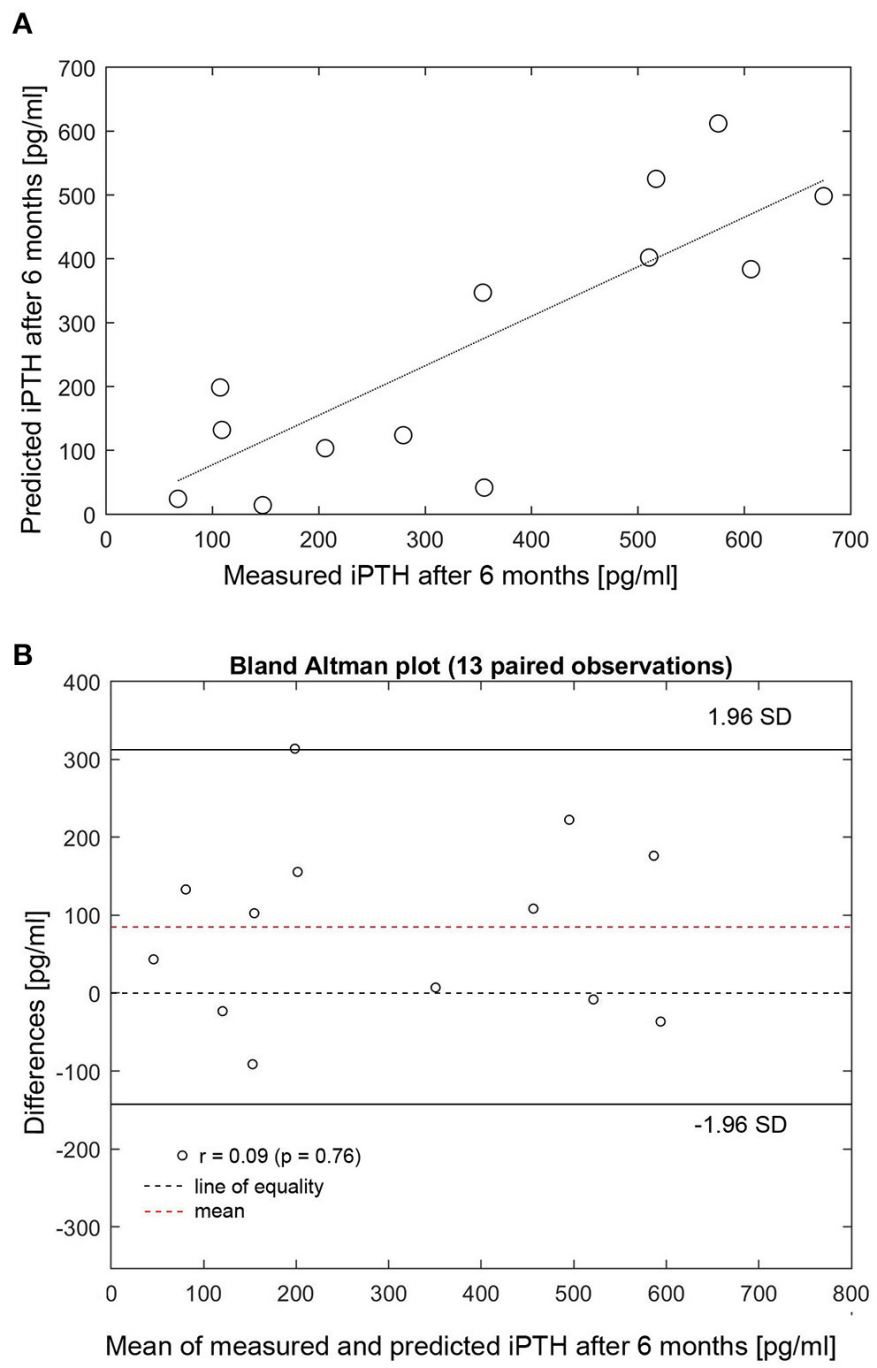
**(A)** Correlation between measured and predicted 6-months iPTH (*n* = 13). (**B)** Bland Altman plot for differences between measured and predicted 6-months iPTH. **(A)** Measured (x-axis) and predicted (y-axis) 6-months iPTH (r = 0.848, *p* < 0.001). HD, hemodialysis; iPTH, intact parathyroid hormone; SD, standard deviation.

The individualized PTG model was used to predict long-term iPTH dynamics based on changes of either iCa or phosphate in 26 patients. Two patients were arbitrarily chosen to present 6-months iPTH predictions based on different clinical scenarios, specifically a 25% rise or fall of phosphate or 10% rise or fall of iCa. The PTG model predictions are shown together with the corresponding observed levels of iCa, phosphate, and iPTH (Figures 3A,B). While iPTH increased over time in patient 1 (Figure 3A), it decreased in patient 2 (Figure 3B). The main difference between these two patients is the iCa trend over time: In patient 1, iCa declined by an average of 10% and measured and predicted iPTH are close (in Figure 3A, the dashed blue line is based on a 10% iCa decline). In patient 2, mean iCa increased over time; again, measured and predicted iPTH levels are close (in Figure 3B, the solid blue line is based on a 10% iCa rise). The depicted predictions consider only the impact of the change of either phosphate or iCa while the other electrolytes are kept constant. Long-term iPTH predictions were then validated by using available iCa and phosphate measurements over 6 months. The correlation between measured and predicted 6-months iPTH is high (r = 0.848, *p* < 0.001, *n* = 13) (Figure 4A). The Bland Altman plot for differences between measured and predicted 6-months iPTH showed no proportional bias (r = 0.09, *p* = 0.76, upper and lower limits of agreement are 142.6 and 312.5 pg/ml, respectively) (Figure 4B).

## Discussion

Our study reveals two important insights: First, a personalized mathematical model of PTG biology can accurately predict short-term, patient-specific peridialytic iPTH responses to diffusive Ca loading; second, the model can predict iPTH trends over 6 months in individual patients. The personalized PTG gland model requires two essential components, a generic (i.e., non-personalized) model of PTG biology which we have recently published and readily available clinical data, such as iCa and phosphate measurements, iPTH history, dialysis vintage, and information about vitamin D therapy to estimate patient-specific model parameters.

Management of sHPT is among the top priorities of precision medicine approaches in HD patients; however, available evidence is based only on studies of dialysis populations (11). The complex biology of PTG during CKD progression is reflected by ambiguous clinical situations. For example, incident dialysis patients with mildly elevated iPTH levels (suggestive for early-stage sHPT) are unresponsive to PTH lowering drugs (i.e., vitamin D and its analogs, phosphate binders; calcimimetics) with the subsequent need for parathyroidectomy or maintenance dialysis patients with persistently high iPTH levels (suggestive for advanced sHPT) respond well to pharmacologic treatment. In this regard, several genetic variants of genes related to VDR and CaSR and Ca and phosphorus transport have been recently identified that may, in part, account for the heterogenous clinical phenotypes, including diverse responses to drug therapy (14).

Current Kidney Disease: Improving Global Outcomes (KDIGO) guidelines recommend iPTH target levels of 2-9 times the upper limit of normal for the assay, reflecting the increased risk with both low (adynamic bone disease) and high (high bone turnover disease) PTH levels (1). A recent Dialysis Outcomes and Practice Patterns Study (DOPPS) analysis demonstrated that in more than two thirds of incident HD patients with initial iPTH levels above 600 pg/ml, the levels decrease to KDIGO targets in the first year on dialysis. However, despite PTH-lowering medication, about 30% of these patients do not achieve target levels. High PTH at dialysis initiation predicted higher PTH levels at 1-year follow-up despite higher doses of PTH-lowering medication (15). These findings highlight the relevance of iPTH history and dialysis vintage for the estimation of patient specific model parameters, i.e., individualization of the PTG model.

The pharmaco-epidemiological Étude PHarmaco Épidémiologique de l'hYperparathyroïdie secondaire en Lorraine (EPHEYL) study, a 2-year, open-cohort, prospective, observational study on incident sHPT, identified four distinct iPTH trajectories: (i) A “rapid PTH drop” group with iPTH decrease over weeks; (ii) a “gradual PTH decrease” group with iPTH decrease over months with mildly elevated phosphate levels; (iii) a “slow PTH decrease” group with iPTH decrease over months with high phosphate levels; and (iv) a “uncontrolled sHPT” group with high PTH and phosphate throughout the study. Most interestingly, the total length of PTH-lowering medication prescribed did not significantly differ between these groups (16). Thus, factors other than PTH-lowering medication most likely account for individual differences in the evolution of iPTH over time. Individual variations in PTG cell proliferation pattern (polyclonal; monoclonal) and in expression and genetics of VDR and CaSR call for novel approaches to predict short- and long-term iPTH kinetics. While previous studies exclusively investigated associations of clinical parameters with population-level iPTH dynamics, we utilized computational and mathematical analysis and modeling of the PTG to make patient-specific predictions of short- and long-term iPTH dynamics. In this study we used the generic PTG model and applied it to real-world data from maintenance HD patients from a previous iCaMB study (13). We were able to validate the individualized model parameters by using short-term changes of iCa as well as long-term iCa and phosphate changes. Moreover, we found that our model allowed 6-months iPTH predictions which closely matched measured levels of iPTH in individual patients.

The limitations of our study are the small sample size and the infrequent iPTH measurements. A single iPTH value prior to diffusive Ca loading might not be representative for current PTG status in general; iPTH measurements in the days before iCaMB assessment would most likely improve model individualization. Measured iPTH values might not fully agree with iPTH prediction curves as the latter consider only changes of either phosphate or iCa while the other electrolytes are kept constant. Two out of 28 patients took cinacalcet prior to iCaMB assessment and were thus excluded. Cinacalcet effectively reduces iPTH levels (17–19) and alters PTG biology [(20, 21)]. The current PTG model cannot be individualized to these patients. Nevertheless, our PTG model can predict iPTH control in response to certain therapeutic interventions, such as phosphate lowering in calcimimetic-naïve patients. Based on HD vintage, iPTH history, phosphate and iCa values as well as information about calcitriol therapy a PTH risk predictor tool can be developed and implemented. Future model iterations may integrate the effect of calcimimetic drugs on PTG model parameters which will allow estimation of the effect of this PTH-lowering intervention on a patient level.

In summary, this is the first report showing that a physiology-based mathematical model of PTG biology can predict short- and long-term iPTH levels in maintenance HD patients. Prospective studies are warranted to understand the utility of this precision approach on sHPT management.

## Data Availability Statement

The raw data supporting the conclusions of this article will be made available by the authors, without undue reservation.

## Ethics Statement

The studies involving human participants were reviewed and approved by Innsbruck Medical University Ethics Committee (protocol number AN2014-0313 343/4.7 391/5.3). The patients/participants provided their written informed consent to participate in this study.

## Author Contributions

MP and GS-T: research idea, study design, and statistical analysis. MP: data collection. MP, DB, PK, and GS-T: data analysis/interpretation and manuscript preparation, drafting, and approval of the final version. All authors contributed to the article and approved the submitted version.

## Funding

The Austrian National Bank supported this work by providing two educational research grants (OENB Jubiläumsfonds Project No. 15366 and 18362) to MP. The funding institution had no role in study design, data analysis and interpretation, preparation or decision to submit the manuscript for publication.

## Conflict of Interest

DB reports having consultancy agreements with Amgen, Ardelyx, Relypsa/Vifor/Fresenius, Sanifit, Sanofi, and Tricidia; serving as a scientific advisor for, or member of, Amgen, Ardelyx, Sanifit, Sanofi, Tricidia, and Vifor/Relypsa; receiving honoraria from Amgen, Ardelyx, Sanifit, Sanofi, Tricidia, and Vifor/Relypsa; having stocks or options in Amgen and Tricida; receiving research funding from National Institutes of Health and Renal Research Institute; serving on the speakers bureau for Sanofi; and being employed by University of Rochester Medical Center. PK is an employee of the Renal Research Institute, a wholly owned subsidiary of Fresenius Medical Care. PK holds stock in Fresenius Medical Care. The remaining authors declare that the research was conducted in the absence of any commercial or financial relationships that could be construed as a potential conflict of interest.

## Publisher's Note

All claims expressed in this article are solely those of the authors and do not necessarily represent those of their affiliated organizations, or those of the publisher, the editors and the reviewers. Any product that may be evaluated in this article, or claim that may be made by its manufacturer, is not guaranteed or endorsed by the publisher.
