# Supplementary material for: Personalized Prediction of Short- and Long-Term PTH Changes in Maintenance Hemodialysis Patients
Source: Front Med (Lausanne). 2021 Sep 14;8:704970. doi: 10.3389/fmed.2021.704970 (PMC8477020; doi:10.3389/fmed.2021.704970)
Supplement: Supplementary file 1 [file Table_1.docx]

Supplementary Material

# Mathematical model of parathyroid gland (PTG) biology: Optimization algorithm

The mathematical parathyroid gland (PTG) model is described in detail elsewhere (Schappacher-Tilp et al, 2019). The model describes PTG biology and its adaptation mechanisms. These mechanisms include the decrease of intracellular PTH degradation rate, the increase in PTH synthesis rate, and the increase in cellular proliferation (Fig. S1). PTH release as well as PTG adaptation mechanisms are regulated by the activation of the calcium sensing receptor (CaSR) and vitamin D receptor (VDR) and their down-stream signaling (Fig. S2). In the mathematical PTG model, this physiological property is reflected in the following sense: the response of the PTG to different ionized calcium (iCa) and phosphate concentrations depends on the stimulus of the CaSR and VDR with regard to the individual optimal iCa ($C_{opt})$and phosphate $(P_{opt})$ values.

While general values for the model are presented in (Schappacher-Tilp et al., 2019), we aim at individualizing key model parameters in order to allow for patient specific short and long-term predictions. Specifically, we choose a set of five parameters, i.e. dialysis vintage as well as calcitriol therapy and all available iCa, phosphate and iPTH concentrations within the preceding six months, that predict iPTH concentration at the time of calcium mass balance assessment. The core of the optimization algorithm is finding reasonable estimates for the individual optimal iCa and phosphate values as well as PTG gland size, intracellular degradation rate, PTH production rate, and PTH clearance rate. While the stimulus of the VDR depends on the optimal calcitriol concentration, the stimulus is less sensitive to deviations from the optimal values. Therefore, we assume a constant optimal calcitriol concentration of 37.5 ng/ml. Since there were no calcitriol measurements available in the laboratory database, we roughly estimate calcitriol levels based on the medical record.

It is worth noting that without restrictions, the parameters are not identifiable, i.e., different combinations of parameters might lead to the same PTH value at the time of the calcium mass balance assessment. However, there are reasonable assumptions described below that restrict the sets of eligible parameters.

First, we estimate the PTH release rate ($r$), number of secretory cells ($S$), intracellular degradation rate $(k_{d}$), and production rate ($k_{p}$) by calculating the steady state of the PTH core model, i.e.

$$0=k_{p}\cdot S-r\cdot PTG-k_{d}\cdot PTG,$$

$0=r\cdot PTG-k_{cl}\cdot PTH$,

under the following restrictions:

The basal rate of $r is$ $B=$0.06 pmol/h, the maximal value $A=$7.2 pmol/h (Shrestha et al., 2010). The basal production rate is $3.96\cdot{10}^{3}$ (Shrestha et al., 2010). The production rate can increase by maximal two-fold. The basal intracellular degradation rate is 0.72 pmol/h, and it can decrease to a half of this value (Brown et al., 2015).

The basal clearance rate is 37.92/h (Shrestha et al., 2010). We assume that the clearance rate is reduced by at least 25% and cannot be reduced by more than 85%. The minimum clearance rate due to clearance by the liver could be adjusted in case of hepatic diseases.

The (normalized) number of cells in the secretory active state at a healthy steady state $\left( S_{0} \right)$is 0.19344. If the average of the last iPTH measurements before the ionized calcium mass balance (iCaMB) assessment was smaller than 150 pg/ml, we assume that the growth of $S$ is not larger than 20%. If the average of the last three iPTH before the iCaMB assessment is larger than 300 pg/ml, the upper bound of $S$ is $S_{0}\cdot\max\left( 2, e^{V\_int \cdot k_{e}} \right),$where $k_{e}=ln(2)/(365\cdot3)$ is based on the assumption that the doubling time of the (untreated) PTG is three years. $Vint$ is the number of days the patient has been on dialysis before the iCaMB assessment. Else, the upper limit equals $S_{0}\cdot max \left( 2, {min(3, e}^{Vint\cdot k_{e}}) \right)$. The lower limit corresponds to the steady state. Only exception is an extended dialysis vintage of at least 5 years. In this case, it is reasonable to assume that the PTG gland has grown, and we set the lower limit equal to $2\cdot S_{0}$.

Based on the iCa concentration at the start of the iCaMB assessment ($C)$and the PTH release rate, we can calculate the individual setpoint $C_{1}$ due to the relationship

$$r=B+(A-B)\frac{1}{1+\left( \frac{C}{C_{1}} \right)^{m}}$$

However, it is not the actual iCa that governs PTH release, but the sensed iCa concentration. Due to the model structure, the sensed iCa concentration cannot be less than 83% of the actual iCa concentration. Therefore, the real setpoint $C_{opt}$ will be between $C_{1}$and $C_{2}$, where $C_{2}$ is defined as

$$r=B+(A-B)\frac{1}{1+\left( \frac{0.83\cdot C}{C_{2}} \right)^{m}}$$

We assess whether iPTH tended to rise or falls over time before the iCaMB assessment by calculating Kendall’s tau. In case iPTH rises significantly, we use the following constraints for $C_{opt}$ and $P_{opt}$:

$C_{opt}$: the lower bound of $C_{opt}$ equals $0.98\cdot C_{2}$ while the upper-bound equals $1.02\cdot C_{1}$.

$P_{opt}:$(1) Phosphate at the time of the iCaMB assessment is smaller than at the beginning of the measurements, i.e., due to dietary restrictions or phosphate binders. We assess this case by calculating the minimum of phosphate of 1/6^th^ of the measurements before the iCaMB assessment. If the mean value of 3 measurements around the minimum is smaller than the mean value of the first three phosphate measurements, the lower bound for $P_{opt}$ is $0.95\cdot min(P_{opt})$, the upper bound is $max(1.1\cdot\min\left( P_{opt} \right), P_{0})$, where $P_{0}=25\%$percentile of the all pre-iCaMB assessment study measurements.

(2) If (1) is not the case, the lower bound of $P_{opt}$ is the 95% of the minimum of all phosphate measurements and the upper bound is the maximum of the average of the first 4 measurements and $P_{0}$.

In case iPTH does not rise significantly (i.e., either p>0.05 or the correlation coefficient is negative), we use the following constraints for $C_{opt}$ and $P_{opt}$:

$C_{opt}$: the lower bound for $C_{opt}$ equals $0.98\cdot C_{2}$ while the upper-bound equals $1.1\cdot C_{1}$.

$P_{opt}$: the lower bound for $P_{opt}$ is 98% of the minimum of all phosphate measurements, the upper bound equals the maximum of 150% of the minimum of all phosphate measurements and $P_{0}$.

The system of ordinary differential equations (ODEs) describing the PTG biology can be written in the following form:

$$\frac{dy}{dt}=A(t,y,C_{opt},P_{opt}, k_{d},k_{p},k_{cl})$$

$\Lambda$ denotes the subset of parameters fulfilling all constraints described above. We assume (pseudo) steady state at the time of the iCaMB assessment.

We set $t=0$ at the time of the iCaMB assessment and solve the following optimization problem:

$\min_{C_{opt},P_{opt}, k_{d},k_{p},k_{cl}, S}\left\| A(0,y,C_{opt},P_{opt}, k_{d},k_{p},k_{cl}) \right\|$ subject

to $\left\{ \begin{matrix} C_{opt},P_{opt}, k_{d},k_{p},k_{cl}, S\in\Lambda\\ \left| PTH_{calc}-PTH_{ref} \right|\leq2\% \end{matrix} \right.$

We can include a quality check of the optimization by calculating the norm of A with the optimized parameters. If this norm exceeds a pre-defined threshold, we cannot find a pseudo steady state under the given constraints.

We use all available iCa and phosphate concentrations 6 months prior to iCaMB assesssment. Concentrations of iCa smaller than 1 mmol/l and phosphate concentration smaller than 0.9 mmol/l were eliminated as input variables for the optimization algorithm. Moreover, iCa and phosphate concentrations smaller than 1 mmol/l were eliminated for the validation process, i.e., iPTH predictions for the 6-months period after iCaMB assessment.

**2 Supplementary Figures**


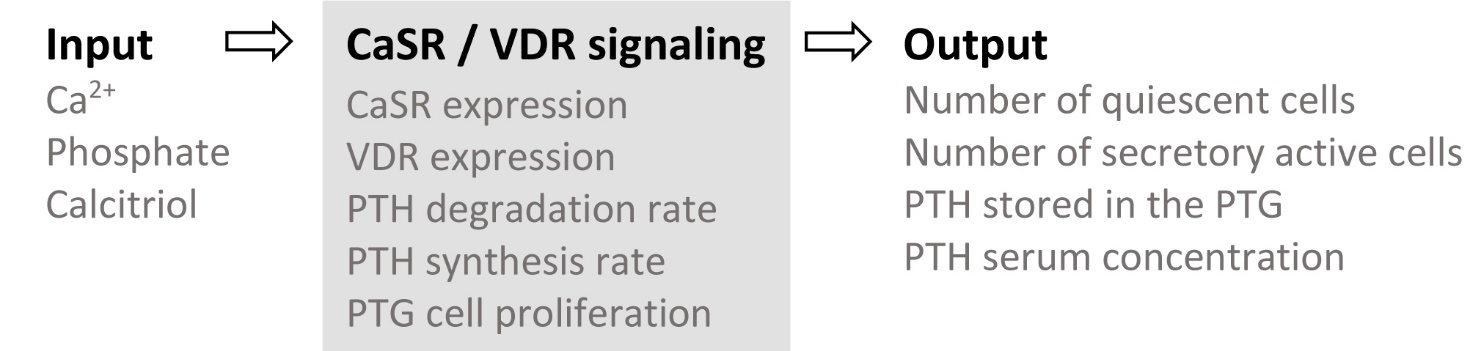


Fig. S1: Block diagram of the generic mathematical PTG model (Schappacher-Tilp et al, 2019). The input parameters are iCa and phosphate concentrations as well as estimated calcitriol concentrations based on calcitriol therapy information. The output of the dynamical model comprises the number of PTG cells, stored PTH, and serum PTH concentration.

Fig. S2: Downstream signaling of calcium sensing receptor (CaSR) and vitamin D receptor (VDR). The effect of phosphate on the CaSR is omitted for clarity.


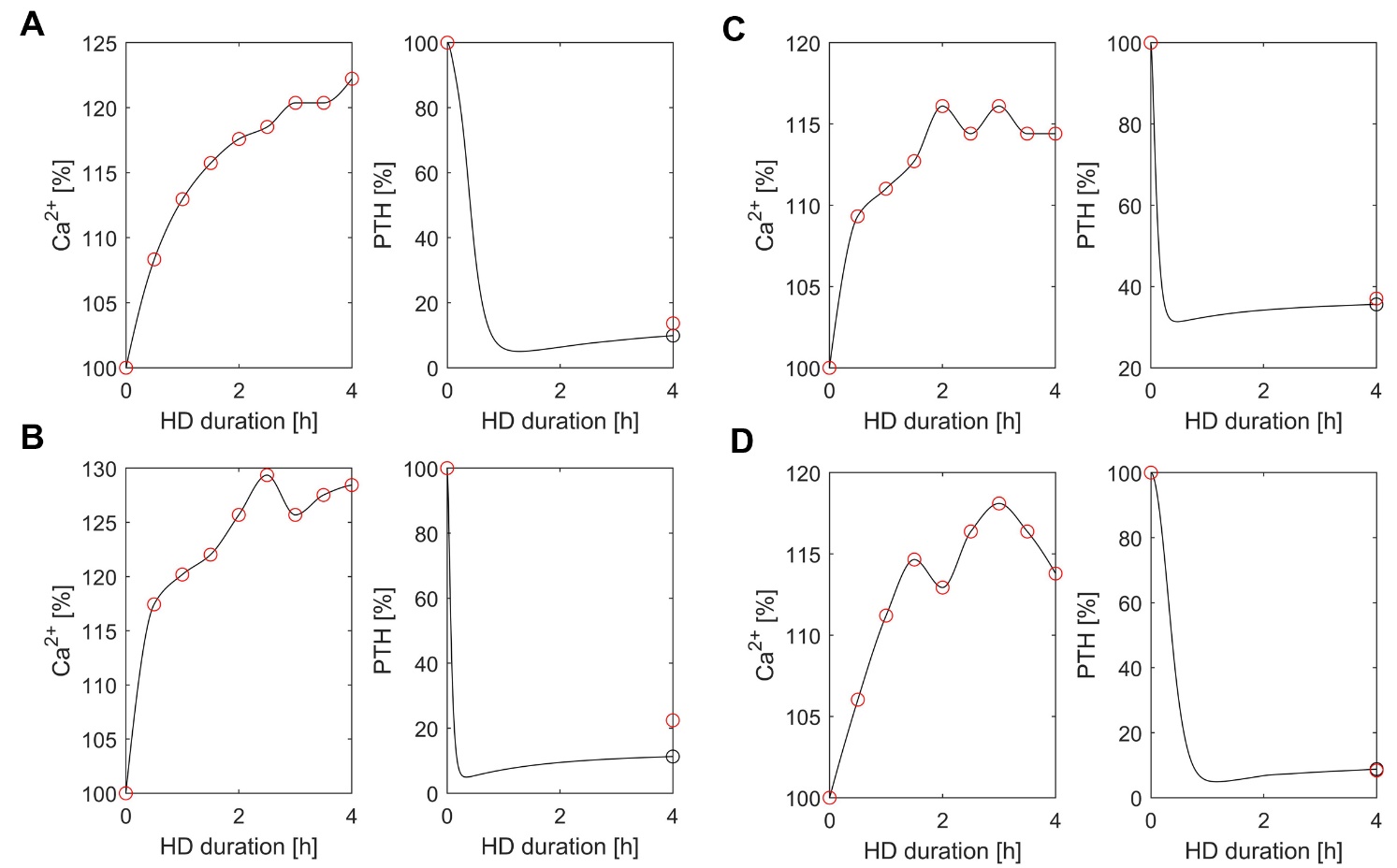


Fig. S3: Relative decrease in iPTH concentration during calcium mass balance assessment of the two exemplar patients (A and B) presented in Fig. 1. In addition, we present the relative decrease in iPTH concentration of two additional exemplar patients (C and D) to show that the same relative increase in iCa might not result in the same relative iPTH decrease.


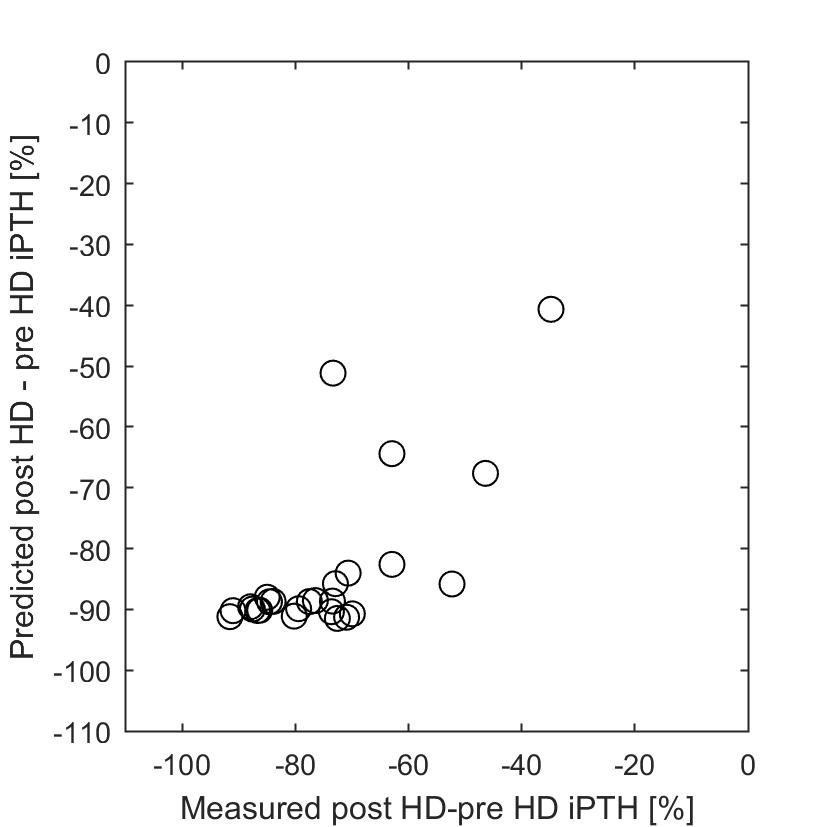


Fig. S4: Measured vs. predicted relative iPTH decrease (post-pre HD, n=26).

**3 References**

Brown EM. Control of parathyroid hormone secretion by its key physiological regulators. In: Bilezikian JP, editor. The Parathyroids. 3rd ed. Waltham, MA: Academic Press/Elsevier (2015). p. 101-18.

Shrestha RP, Hollot CV, Chipkin SR, Schmitt CP, Chait Y. A mathematical model of parathyroid hormone response to acute changes in plasmaionized calcium concentration in humans. *Math Biosci.* (2010) 226:46–57. doi: 10.1016/j.mbs.2010.04.001
